# Second hit in cervical carcinogenesis process: involvement of wnt/beta catenin pathway

**DOI:** 10.1186/1755-7682-1-10

**Published:** 2008-07-07

**Authors:** Carlos Perez-Plasencia, Alfonso Duenas-Gonzalez, Brenda Alatorre-Tavera

**Affiliations:** 1 Unidad de Investigación Biomédica en Cáncer, Instituto de Investigaciones Biomédicas, Universidad Nacional Autonóma de Mexico UNAM, Instituto Nacional de Cancerologa INCAN, Mexico City, Mexico; 2 Laboratorio de Oncología Genómica, Instituto Nacional de Cancerologa INCAN, Mexico City, Mexico; 3 UAM-Xochimilco, Departamento de atención a la Salud, Calzada del Hueso, No. 1100, Col, Villa Quietud, 04960, Mexico City, Mexico

## Abstract

The Human papillomavirus plays an important role in the initiation and progression of cervical cancer. However, it is a necessary but not sufficient cause to develop invasive carcinoma; hence, other factors are required in the pathogenesis of this malignancy. In this review we explore the hypothesis of the deregulation of wnt/β-catenin signaling pathway as a "second hit" required to develop cervical cancer.

## Background

### HPV and cervical cancer

Cervical carcinoma (CC) is one of the most common cancers and a leading cause of death among women worldwide. [1]. The number of patients who died from this disease in 2000 was estimated at >200,000 [2]. An important percentage of cases occur in the developing world, where in some countries CC is the main cause of death in reproductive-aged and economically active women with limited access to early diagnosis or effective treatment [1].

Over 30 years ago, some authors pointed out that CC had sexually transmitted disease behavior [3], while others hypothesized and analyzed a possible role of the human papillomavirus (HPV) in this neoplasia [4,5]. But it was until November 1991 that the association between HPV infection and CC was officially established, after considering the epidemiologic and molecular evidence that the DNA of HPV is integrated in more than 99% of cervical carcinoma specimens [6,7].

At present, more than 100 types of HPV have been identified [8], 40 of which infect the genital epithelia. Genital HPVs are classified in three groups according to their potential to induce cervical lesions: high-risk, probable high-risk and low-risk types [9]. Between high-risk viral types, 16 and 18 HPV types are the most frequent viral types, with nearly one half of all cervical cancers the former, and 15%, the latter [9]. Fortunately, not all patients infected with oncogenic HPVs will develop CC due to frequent spontaneous clearance of viral sequences. This discrepancy indicates that the majority of HPV infections are sub-clinical; therefore, only a small number of oncogenic HPV infections will produce early epithelial lesions, and only a very small number of these lesions will lead to CC [10,11]. Hence, infection with high-risk HPV is a necessary – but not sufficient – cause for developing cervical carcinoma; thus, other types of factors such as cellular, immunological, genetic, epigenetic, environmental, etc. can affect the final outcome of the disease. In this context, viral factors have been reasonably explored, producing adequate evidence to postulate the occurrence of three events during HPV course infection as cancer promoters: viral DNA integration to host genome; expression of viral oncoproteins E6 and E7, and finally the complex interactions between E6/E7 and cellular proteins. In this carcinogenesis complex model, identification of viral, host, and environmental factors exerting an influence on the risk of disease progression from early cervical abnormalities to invasive cancer will lead us to better knowledge of the natural history of HPV infection.

**Figure 1:**
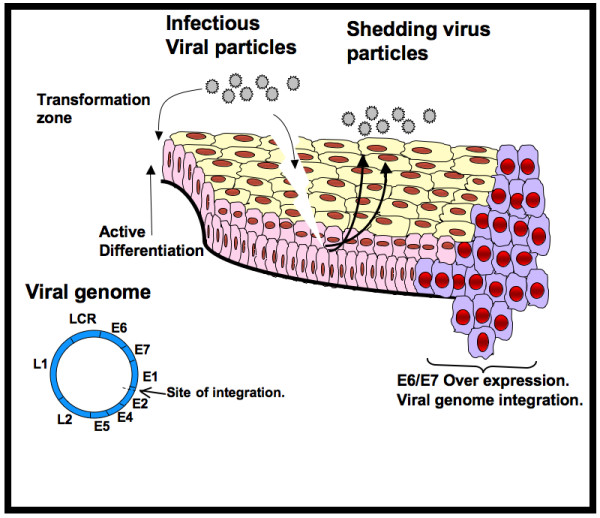
**HPV viral cycle and cervical cancer development**. Human papillomavirus (HPV) gains access to basal cells through microabrassions or by infecting the transformation zone, an abrupt transition from a columnar to a squamous epithelium. Infected cells actively express the early genes E1, E2, E4 and E5. Viral oncoproteins E6 and E7 are expressed in limited amounts due to transcriptional repression exerted by E2. Infected basal cells migrate to the lumen as they differentiate; differentiated epithelial cells express the late capside genes L1 and L2. In subclinical infections or low grade intra-epithelial-lesions (LGSIL) the viral genome replicates as an episome and is encapsidated in the nucleus of the upper layer epithelium cells. Shed viral particles then can infect new zones of epithelium or be sexually transmitted. Only a limited number of infections progress to high grade intra-epithelial-lesions (HGSIL) and cervical carcinoma (CC). The progression of LGSIL to CC is associated with the integration of the HPV genome into the host genome and the loss of transcriptional repression exerted by E2.

Once HPV has infected basal cells, the viral genome is replicated actively as episome and early genes (E1–E7) are expressed. E1 and E2 are important proteins for viral genome replication and viral cycle completion [12]. E1 plays an important role in the maintenance of the viral genome as episome [13]. In turn, E2 is involved in the negative regulation of the transcriptional activity of viral oncogenes E6 and E7 [14]. However, E5, E6, and E7 are required to increase basal-cell proliferation leading to an increase of the viral genome replication rate; therefore, a limited expression of these genes can take place during early stages of infection [15]. Late genes (L1 and L2) encode viral capsid proteins and are expressed during the late stages of virion assembly in middle and upper epithelium layers. Finally, virions are encapsidated and shed into the genital tract [16] where they can infect other areas of epithelia or be sexually transmitted (Figure 1).

An important step during HPV infection corresponds to viral integration into the host genome. The HPV genome is usually replicated as episome or extrachromosomic molecule in benign cervical precursor lesions. Cancer tissues can contain both episomal and integrated HPV DNA [17]. Because the HPV genome is a ring molecule, it requires being open in order to be integrated; this process often breaks the E1–E2 open reading frames. Part of E2 and adjacent regions to E2–E4, E5, and L2 are commonly deleted after integration; therefore, the partial transcriptional repression exerted by E2 is lost and viral oncogenes E6 and E7 are actively expressed in CC tissues [18]. Furthermore, viral integration frequently occurs in chromosomic regions where genes involved in tumorogenesis, such as *MYC, NR4A2, hTERT, APM-1, FANCC*, and *TNFAIP2*, etc., are found [19].

E6 and E7 epithelial expression and its interactions with cellular proteins have been at the center of the HPV biomedical research scenario probably for the past 20 years. The central core of the classic E6/E7 model is the binding and inactivation of tumor suppressor proteins p53 and pRb respectively; which was established between the late 1980s and the early 1990s [20,21]. Currently, it is well-known that E6 and E7 interact with a plethora of cellular proteins that participate in molecular pathways involved in the activation and establishment of the malignant phenotype.

One of the candidate signaling pathways that has been recently studied in cervical carcinoma model is the Wnt/beta catenin pathway. The role of wnt signaling in cancer was initially described 20 years ago with the seminal discovery of wnt-1 gene as an integration site for mouse mammary tumor virus (MMTV) [22]. Since then a wealth of information has highlighted the role of the wnt pathway in the control of several biological processes, for instance, cell fate specification, proliferation, migration, cell adhesion, cell polarity, tissue architecture, and organogenesis [23,24]. In an adult body, Wnts regulate hematopoiesis, osteogenesis, angiogenesis, and adipogenesis [25-27]. This wide range of biological effects shows a high pleiotropism of wnt signals, which are also involved in human diseases including cancer. Several reports have demonstrated aberrant activation of the wnt signaling pathway in different human cancers, including colorectal [28]; gastric [29] and melanoma [30]. In CC little is known about the wnt signaling pathway, however we and other researchers have recently described alterations on this pathway in cervical neoplasia. The aim of this review therefore, is to analyze current evidence of wnt pathway involvement on CC.

### Wnt/beta-catenin signaling pathway

Wnt binds to seven-transmembrane domain proteins denominated Frizzled (FZD). The broad range of cellular processes regulated by the wnt pathway can be explained -at least in part-by the high diversity between wnt proteins and FZD receptors. In the human genome, 19 WNT and 11 FZD genes have been identified. For a complete list of WNT and FZD genes, please visit the World Wide Web Wnt Homepage [31]. Interactions between Wnt proteins and their receptors show an important rate of promiscuity [32]; therefore, a Wnt protein can bind with different FZD receptors, and vice versa. This interaction requires the cooperation of LRP5 and LRP6, which are long single-pass transmembrane proteins acting as co-receptors [33]. Mutations in both genes can lead to developmental defects similar to inactivating mutations in individual Wnt genes, e.g., defects in dorsal thalamic development, skeletal and neural tube abnormalities, decrease in osteoblast proliferation, osteopenia, and persistent embryonic eye vascularization [34-36]. This evidence shows that LRPs proteins perform an important role in Wnt pathway activation and regulation. The interaction between LRP5/6, FZD, and Wnt can be regulated by secreted proteins such as Dikkopf (Dkk), secreted frizzled-related proteins (sFRP), and Cerberus1 (Cer1), which can inhibit wnt signaling through direct binding to wnt or co-receptor molecules. Dkk binds to LRP with other transmembrane proteins, the Kremens (Krm); thus promoting LRP internalization and inactivation [37,38]

**Figure 2:**
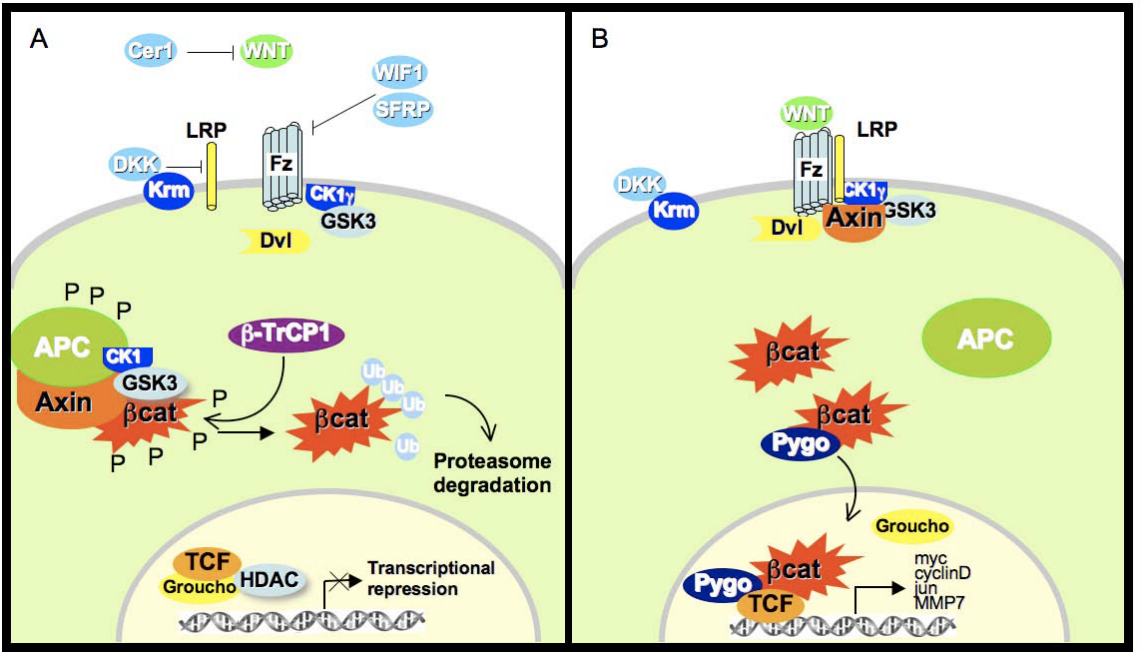
**Wnt canonical signaling pathway**. A. When the wnt signaling pathway is not active, β-catenin is bound to the degradation complex composed by APC, axin, and the serine/theronine kinases CK1 and GSK3. The main role of the degradation complex is to phosphorylate β-catenin leading to its degradation by means of the proteasome-ubiquitin pathway. There are several negative regulators that operate at the receptor-ligand level; such as, Cer1, DKK, WIF1 and sFRP, whose function is to modulate positive signals induced by Wnts. B. The contact of wnt with its receptors leads to the stabilization of β-catenin and its accumulation in cytoplasm and nucleus. β-catenin displaces the transcriptional repressor groucho from the LEF/TCF complex, leading to the activation of target genes, such as c-myc and cyclinD1, which are involved in cell proliferation and cell cycle progression.

Currently there are three well-known pathways that are activated after Wnt couples with its receptors. The pathway which is activated depends on the specificity of the Wnt ligand and its FZD receptor and surely on cellular components such as co-activators or co-repressors that exert a fine-tune regulation. These pathways are: 1. The canonical pathway which induces the stabilization and entry to nucleus of β-catenin, where it affects the transcription of target genes (figure 2) [39]. 2. The planar cell polarity pathway (PCP) which establishes the polarization of cells within the plane of a cell sheet. This pathway is important during neural tube closure and cochlear extension [40]. And finally, 3. The Wnt/Ca+2 or "non-canonical pathway", which regulates cell adhesion and motility mediated by the Wnt-5a, triggers intracellular Ca^2+ ^release in order to activate Ca^2+^-sensitive enzymes such as the protein kinase C (PKC) and Ca^2+^/calmodulin-dependent kinase II (CaMKII) without the activation of the β-catenin pathway [41]. The canonical pathway is the best understood pathway, a hallmark of cancer and a central topic in this review.

Following the trimerization of Wnt/Fzd/LRPs, the LRP co-receptor is phosphorylated leading to the binding and phosphorylation of Disheveled (Dvl), which transduces the Wnt signal into the cell through direct binding with FZD [42,43]. Dvl is a central player in the Wnt signal routing and amplification through pathway-specific effectors [44]. Dvl interacts with axin, which performs a scaffolding function in the wnt pathway by its association with key proteins for β-catenin phosphorylation and poly-ubiquitination, including GSK-3β, CK1, APC, and β-catenin itself. The consequence of axin phosphorylation is β-catenin activation through axin sequestration and its inactivation by the proteasome pathway. Some reports indicate that in mammalian cell culture wnt activation precedes axin degradation, however, the exact mechanism remains unclear [45].

An important hallmark in the canonical Wnt pathway is the stabilization of β-catenin in cytoplasm and its accumulation into the nucleus. This is possible due to the detachment of β-catenin from the degradation complex, a multi-protein assembly activated in the absence of wnt signaling, whose main task is to add ubiquitins to β-catenin resulting in its inactivation by means of the ubiquitin-proteasome pathway [24]. In an unstimulated cell, β-catenin interacts with E-cadherin and α-catenin at epithelial cell adherens junctions, regulating cell adhesion. The excess of β-catenin levels are modulated by means of the degradation complex [46]. A key component of the degradation complex is APC. When β-catenin binds to APC, it displaces the union of axin because the binding affinity of β-catenin increases dramatically upon phosphorylation and because the binding motifs of APC to axin and β-catenin overlap [47]. Most colorectal tumors contain truncating mutations on APC, which leads to an inability to bind Axin or degrade β-catenin [48].

After the FZ/LRP activation by its ligand, the degradation complex kinase activity is inhibited. Consequently, non-phosphorylated β-catenin is stabilized, and increasing levels tend to accumulate in the cytoplasm. β-catenin accumulation in cytoplasm leads to its nuclear accumulation, where it is associated with lymphoid enhancer-binding factor 1/Tcell-specific transcription factor (LEF/TCF) and transcriptional activator Pygopus (Pygo). Pygo contains a domain (PHD), which is shared by many nuclear proteins with a role in chromatin remodeling and transcriptional co-activation [49]. Mutations in Pygo result in many defects that are very similar to those of wnt loss-of-function [50].

In the absence of wnt signaling, TCF is bound to Groucho, forming a repressor complex of Wnt target genes. Groucho can repress gene transcription by inhibition of basal transcriptional machinery and recruitment of histone deacetylases (HDACs) [51,52]. Although Groucho does not interact with DNA, it exerts its repressive function by its interaction with the tails of core histones H3 and H4 and by modifying chromatin structure [53]. Thus, the specificity of the genes that will be repressed by Groucho is provided by LEF/TCF.

When β-catenin enters the nucleus, it converts the repressor LEF/TCF/Groucho into a transcriptional activator complex. This is possible due to Groucho displacement by interaction between β-catenin and LEF/TCF, thereby activating wnt target genes [54]. Several genes activated by the wnt signaling pathway, which are mainly involved in cell proliferation and differentiation processes, have been identified; for a comprehensive list of genes regulated by this signaling pathway, please visit the Wnt homepage.

### Wnt pathway and cervical carcinoma

To this point we have reviewed how the wnt/β-catenin pathway turns on complex signaling events leading to the activation of target genes. Mutations in several components of this pathway have been studied and identified in nearly all human cancers. However, in cervical cancer only few studies have shown the involvement of certain wnt/β-catenin genes in their pathogenesis. In this regard, it has been suggested that HPV-immortalized human keratinocyte transformation requires a second hit and could be achieved thanks to the activation of the canonical wnt pathway [55]. This hypothesis is supported by the fact that β-catenin expression is increased in 73% of CC specimens, with cytoplasmic and nuclear positive staining, nevertheless mutations were found in only 20% of the analyzed cases [56]. Thus, evidence suggests the activation of β-catenin at an upstream level, which can be accomplished by the inactivation of negative regulators such as APC and axin. It is well-known that during carcinogenesis, distinct tumor suppressor genes are inactivated by abnormal methylation of CpG islands, probably by means of an increase in DNA methyltransferase (DNMT) activity [57]. HPV16/E7 has the capacity to bind and increase the dna methyl transferase enzyme (DNMT1) activity, the enzyme responsible to add methyl groups on CpG islands [58]; thus, it is feasible that negative wnt/β catenin-pathway regulators could be inactivated by methylation. In this respect, sFRPs, axin, DICKKOPF (Dkk) and APC genes have enriched CpG islands in their promoters which can be found as hypermethylated in some neoplasias including CC [29,59-61]; therefore, it is probable that these genes could be inactivated by promoter methylation during cervical carcinogenesis.

Another mechanism involved in the upstream activation of the wnt/β-catenin pathway is over-expression of pathway activators such as wnt ligands, frizzled receptors, and disheveled. There is evidence showing over-expression of WNT10B, -14, FZD10, and DVL-1 in cervical cell lines [62-65]; nonetheless, this has not been explored in pathological specimens by traditional single-gene methodologies such as RT-PCR, immunohistochemistry, or Western and Northern blot.

**Figure 3:**
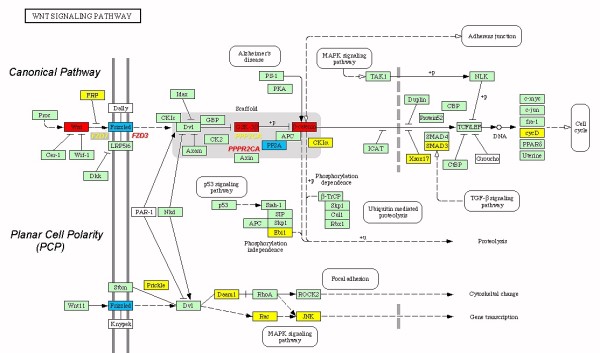
**Wnt signaling pathwhay in cervical cancer**. Expression levels of some genes participating in the wnt/β-catenin signaling pathway are altered in CC. Interestingly, Planar Cell Polarity pathway (PCP) is present in normal epithelial cells but is down-regulated in CC. A possible mechanism to accomplish PCP down-regulation is the inactivation of sFRP transcription. Yellow boxes indicate a down-regulation of expression in tumor cervices' samples compared to normal cervices' samples. Red boxes indicate a higher gene expression in cervical carcinoma samples. Blue boxes show that a member of the indicated gene family is expressed in normal tissues, and another member of the same family in tumors. Green boxes indicate that the gene was not altered. Figure modified from Infect Agent Cancer. 2007; 2: 16.

We recently conducted a genome-wide expression analysis in HPV16 CC compared against normal cervix epithelia [66]. We were able to identify genes and cellular pathways with aberrant expression levels, and one of the most altered pathways was wnt/β-catenin. In this work, we analyzed the expression levels of 55,000 sequences that virtually represent all genes expressed in the human genome. In this way, we showed a significant increment of Wnt4, -8a, Fzd2, GSK3β, and β-catenin in tumors . In addition, genes also belonging to this pathway are actively expressed in normal cervical epithelia, such as sFRP4, PPP2C, and FZD7 (Figure 3). This evidence demonstrates two important facts: First, deregulation in specific genes belonging to wnt/β-catenin pathway could play an important role in cervical carcinogenesis, and second, the presence of some wnt/β-catenin-related genes in normal tissues suggests that this pathway is involved in cervical epithelial differentiation. Additionally, it is noteworthy that sFRP4, a negative regulator of wnt that inhibits receptor activation by competition with wnt proteins, is actively expressed in normal epithelia and absent in CC tissues, which indicates that this gene could be important in balancing the positive signals leading to the binding of wnt to its receptor, hence regulating overactivation in wnt-induced cell signaling.

Interestingly, gene components of the planar cell polarity (PCP) pathway were actively expressed in normal cervices, showing that this branch of wnt signaling is downregulated in CC (Figure 3). In vertebrates, PCP is considered as any process affecting cell polarity within an epithelial plane and involving one or more core PCP genes. PCP has shown to be an important developmental and adult tissue differentiation process. At present, the developmental processes that meet these criteria include convergent extension, neural tube closure, eyelid closure, hair bundle orientation in inner ear sensory cells, and hair follicle orientation in the skin [67]. To our knowledge, there are no previous reports showing active PCP genes in normal cervical epithelia. This result demonstrates that during the carcinogenesis process, infected cervical cells turn off the PCP pathway, activating the canonical pathway with an increase of genes participating in it, e.g., Wnt4, -8A, FZD2, CTNNB1, among others. This turning-on of the canonical pathway leads to the activation of target genes such as MYC, JUN, FOS, and RRAS (for a complete list of altered genes in HPV16CC, please see the additional file 2 in [66]). A possible mechanism explaining how the canonical pathway is privileged in HPV16CC might be sFRP4 inactivation in normal cells. In an unpublished work, we observe that sFRP4 and DKK are inactivated by promoter methylation in CC; when cells are treated with DNMTs inhibitors we detect a re-expression of these genes (unpublished data). It has been shown that the sFRP gene is frequently inactivated by promoter methylation in gastric and hepatocellular cancer (HCC) [29,68]. Moreover, sFRP1 restoration attenuated Wnt signaling in HCC cells, decreased abnormal beta-catenin accumulation in nucleus and suppressed cell growth [68]. As previously described, it is common to find cytoplasmic and nuclear β-catenin accumulation in CC accompanied by occasional mutations in the CTNNB gene. With the previous evidence; we could speculate that canonical-pathway activation in CC is accomplished by means of inactivation of wnt negative regulators such as sFRP genes, and particularly SFRP4 and Dkk, as we were able to show [66,69].

### Concluding remarks

Even though the extensive use of the Papanicolau smear and colposcopy examination have significantly decreased the mortality rates, CC remains as the second cause of death in women worldwide. HPV sequences have been found in more than 99% of CC and HPV infection is the most important etiologic factor in cervical carcinogenesis. Besides, HPV infection is very common among the young sexually active population, but only a small fraction of infected individuals will develop cervical carcinoma later in life. Thus, HPV can be considered as an initial hit in the multistep carcinogenesis that leads to the development of CC. The molecular pathways involved in the progression of HPV-infected cells to CC have not yet been accurately identified. Here, we reviewed the role of Wnt/β-catenin pathway activation and the inactivation of planar cell polarity pathway in CC cells as a second hit to develop CC.

In CC mutations on CTNNB1 gene are uncommon; thus the activation on wnt signalling pathway it seems to be activated upstream of/β-catenin. This fact could be achieved by means of negative regulators inactivation or over-expression of pathway activators.

Other branch in wnt signaling pathway that could be relevant during CC pathogenesis is planar cell polarity (PCP) pathway, which is involved in differentiation processes. PCP is a key differentiation and morphogenetic process involved in development of epithelia. In normal cervical epithelia, cells are polarized and migrate from basal to the luminal space as they differentiate. Interestingly, PCP component genes are down-expressed in CC, indicating that this mechanism could be abated prior the establishment of the neoplasia. From the diagnostic point of view this fact could be important, because if we were able to show as an early process, we could account with potential molecular markers for early detection.

## Competing interests

The authors declare that they have no competing interests.

## Authors' contributions

CPP conceived and wrote the manuscript, ADG and BAT participated in the discussion and analysis of the content.
